# Dynamics of Persistent Submicroscopic and Microscopic *Plasmodium falciparum* in Pregnant Women Under Intermittent Preventive Treatment: A Study Cohort in Benin

**DOI:** 10.1093/ofid/ofae762

**Published:** 2025-01-06

**Authors:** Sayeh Jafari-Guemouri, Robinson Dégbègni, Laura Courtois, Manfred Accrombessi, Achille Massougbodji, Xavier C Ding, Nicaise Tuikue Ndam, Atika Mama, Nadine Fievet, Véronique Sarrasin-Hubert, Gilles Cottrell, Valérie Briand

**Affiliations:** UMR261 MERIT, Université Paris Cité, IRD, Paris, France; Clinical Research Institute of Benin (IRCB), Abomey-Calavi, Benin; Genetics Department, Institut Curie, PSL Research University, Paris, France; Clinical Research Institute of Benin (IRCB), Abomey-Calavi, Benin; Faculty of Infectious and Tropical Diseases, Disease Control Department, London School of Hygiene and Tropical Medicine, London, Royaume-Uni; Clinical Research Institute of Benin (IRCB), Abomey-Calavi, Benin; Abbott Rapid Diagnostics, Baar, Switzerland; UMR261 MERIT, Université Paris Cité, IRD, Paris, France; Clinical Research Institute of Benin (IRCB), Abomey-Calavi, Benin; UMR261 MERIT, Université Paris Cité, IRD, Paris, France; Centre National de Référence du Paludisme, Paris, France; UMR261 MERIT, Université Paris Cité, IRD, Paris, France; UMR261 MERIT, Université Paris Cité, IRD, Paris, France; Epicentre, Paris, France

**Keywords:** intermittent preventive treatment, msp-2 fragment analysis method, *plasmodium falciparum*, pregnancy, submicroscopic

## Abstract

**Background:**

Malaria infections in pregnancy are a major cause of maternal morbidity and neonatal mortality in sub-Saharan Africa. A high proportion of these infections are submicroscopic, which are usually asymptomatic and therefore untreated during pregnancy. Intermittent preventive treatment with sulfadoxine-pyrimethamine (IPTp-SP) aims to prevent and treat all potential infections whether submicroscopic or not. However, the resistance of parasites to SP is steadily increasing. The dynamic of microscopic and submicroscopic infections in a cohort of Beninese women throughout their pregnancy and its relation to IPTp-SP has been assessed.

**Methods:**

As a subsample of the RECIPAL project, 130 women with at least 2 infections detected by polymerase chain reaction during their pregnancy were included. Infections were categorized as new (isolated) or persistent based on *msp-2* genotyping, where persistent infections had identical genotypes in all studied time points. Submicroscopic infections were defined as polymerase chain reaction–positive and thick blood smear–negative. The persistence of infections according to IPTp-SP uptake was assessed.

**Results:**

A total of 73.1% of women (95 women of 130) had exclusively persistent infections throughout their pregnancy, whereas only 7.7% (10 of 130) had exclusively new infections. During pregnancy, the median time spent with 1 persistent infection was 7.2 weeks. A considerable proportion of these persistent infections 64.3% (72 of 113) was only submicroscopic. Approximately 20% of these persistent infections occurred despite the use of IPTp-SP.

**Conclusions:**

Using new antimalarial combinations could contribute to limit the persistence of submicroscopic infections and their probable negative effects on the mother and the fetus.

## Teaser

Intermittent preventive treatment with sulfadoxine-pyrimethamine prevents malaria in pregnant women in Africa. A sensitive genotyping method shows that a considerable number of women have persistent infections throughout pregnancy despite this treatment. Using new antimalarial combinations could contribute to limit this persistence.

Malaria poses a major public health risk to pregnant women living in areas of high *Plasmodium falciparum* transmission [1]. Pregnant women face greater risk of infection and infection-related complications compared to other adults [2]. Malaria in pregnancy (MiP) is a major cause of maternal morbidity (anemia) and poor fetal outcomes such as miscarriage and stillbirth. MiP is associated with fetal growth restriction and preterm birth, contributing to low birth weight in newborns, resulting to an increased risk of neonatal and infant mortality [3, 4]. Approximately 100 000 children die annually in sub-Saharan Africa because of malaria-related low birth weight [4].

In the past decade, several studies have shown a high prevalence of carriage of submicroscopic infections from the introduction of polymerase chain reaction (PCR)–based molecular methods that are more sensitive than the standard malaria detection tools (thick blood smear [TBS] and Rapid Diagnosis Test). This highly sensitive molecular method enlightens the real prevalence of malaria infections, especially in pregnant women [5, 6].

The importance of submicroscopic infections (negative thick smear/positive quantitative PCR) is potentially high because they are suspected to affect women's and newborns' health [6, 7], mostly because they are usually asymptomatic and thus remain untreated during pregnancy. This parasite burden is a reservoir that can play a significant role in the transmission and maintenance of the infection in endemic areas [8].

To prevent the adverse outcomes of MiP in areas of moderate to high malaria transmission of sub-Saharan Africa, the World Health Organization recommends the use of insecticide-treated mosquito nets and intermittent preventive treatment in pregnancy (IPTp) with sulfadoxine-pyrimethamine (SP). IPTp-SP has been recommended by the World Health Organization since 2004 and is adopted by the Benin health system since 2006. The current strategy consists in the administration of monthly curative doses of SP starting in the second trimester of pregnancy with a dose-spacing interval of at least 1 month [9]. SP continues to be the drug of choice for IPTp because it is safe, easy to administer, and efficacious both in experimental situations and real conditions. However, resistance of parasites to SP has been steadily increasing over the past decade [10]. Ongoing evaluations of SP resistance levels and strategy effectiveness regarding both clinical and parasitological outcomes in pregnant women are therefore needed. According to several studies, parasite resistance to IPTp-SP is driven by increasing prevalence of high-level resistant parasite strains, mostly in eastern and southern Africa [11, 12]. The selection of these resistant parasite strains is alarming and thus continues to threaten effectiveness of SP throughout sub-Saharan Africa [13, 14].

In this study, in a cohort of Beninese pregnant women and in a context of existing resistance to SP, we assessed the dynamic of microscopic and submicroscopic infections (in both isolated and persistent infections) throughout the pregnancy and its relation with IPTp-SP. Persistent infection is explained by a parasite population that survive during a defined time through pregnancy, whereas isolated infection occurs once during pregnancy and is replaced by a new parasite population that is genetically different. We used data collected during the “Retard de Croissance Intra-utérin et Paludisme” (RECIPAL) project, a cohort study of malaria in pregnant women followed up from preconception to delivery in Benin from 2014 to 2017. The methodology used for the determination and identification of the nature of the infections was based on the genotyping of the highly polymorphic *msp-2* gene in *P falciparum* [15].

## MATERIALS AND METHODS

### Study Population and Design

This study was approved by the Ethics Committee of the Institut des Sciences Biomédicales Appliquées and the Ministry of Health in Benin. Before recruitment, the study was explained in the local language to each woman and her voluntary consent was obtained. It was performed using blood samples collected during the RECIPAL study conducted in Southern Benin (2014–2017) [16]. Briefly, the RECIPAL study aimed to assess the prevalence and consequences of malaria in the first trimester of pregnancy on maternal and child health. It was based on a cohort of 411 pregnant women who were recruited before conception and then followed monthly from early pregnancy to delivery. In April 2018, 378 of the 411 pregnant women consented to have the samples collected during the RECIPAL study. They were followed up monthly for clinical examination and malaria screening. Women with microscopic malaria were treated with quinine in the first trimester and with Coartem in the second and third trimesters of pregnancy. The maternity staff was encouraged to administer at least 3 doses of IPTp during their pregnancy as recommended by national guidelines [16].

### Malaria Diagnosis

Within 24 hours after blood sample collection, TBS were stained with Giemsa and parasitemia was quantified by the Lambaréné method [17].

Submicroscopic malaria infections were detected by using an ultra-sensitive molecular diagnostic approach using real-time PCR targetting the gene that encodes the 18S unit of *Plasmodium* rRNA [18]. Infections with TBS-negative/PCR-positive results were defined as submicroscopic, whereas those with TBS-positive/PCR-positive were defined as microscopic infections.

### Study Population

Our study population was a subsample of the RECIPAL cohort and included all women who had at least 2 successive PCR-positive infections during pregnancy, occurring within a maximum of 2 months. PCR-positive infections that were detected during scheduled antenatal care visits, unscheduled visits and at delivery were all considered. Based on these criteria, 130 pregnant women with several infections during their pregnancy and until delivery were included in this study.

### Molecular Genotyping of the Polymorphic Gene *msp-2*

In this study, a fragment-analysis method was used for the determination and identification of the nature of the infections and enumerate all of the *P falciparum* genotypes in pregnant women's isolates. This method is based on the polymorphism of the gene encoding for merozoite surface protein 2 (MSP-2), an abundant surface component in the erythrocyte invading stage of *P falciparum* [19]. Analyses of a fluorescent PCR of the highly polymorphic block 3 of the *msp-2* gene were conducted as described in a previous study [15]. This method is based on a PCR amplification with a fluorescent primer, followed by a capillary gel electrophoresis, processed in an ABI Prism 3130 XL Genetic Analyzer (Perkin Elmer Applied Biosystems) to enumerate and quantify fluorescent fragments and therefore to discriminate genotypes of different sizes in each isolate. This method permits to detect much less abundant amplicons compared to standard agarose gel electrophoresis-based methods. The true value of this method has already been evaluated in clinical settings in previous studies [15, 20–22]. The number of *msp-2* genotypes detected at various time points in pregnant women included in this study are summarized in a Supplementary Table (See Supplementary Table).

The ability of this method to differentiate new infections from persistent infections in in vivo studies is more precise in comparison to other molecular methods because the proportion of each genotype is determined in a polyclonal isolate. We considered that 2 successive PCR positive infections were genotypically identical in the 2 following cases: (1) same number of genotypes and same genotypes in both samples and (2) samples different for 1 or 2 minor genotypes (<2%).

The genotyping data showed 2 types of infection: (1) persistent infection: same *msp-2* genotype profile between 2 samples or more or (2) isolated infection: an infection with a different *msp-2* genotype profile compared to those detected at previous and following visits.

The duration of a persistent infection was defined as the time between the 2 collected samples farthest apart infected with the same genotype. For example, in the case of a pregnant woman with 4 visits (V1, V2, V3, and V4) and their corresponding genotypes respectively at each visit: A, B, B, B, we identified an isolated infection at V1 and a persistent infection extending from V2 to V4.

### Data Analysis

For each included woman, the number of persistent and isolated infections throughout pregnancy, as well as the duration of each persistent infection were estimated. Then, the proportion of time of pregnancy spent with persistent infection(s) was calculated. The duration of pregnancy was defined as the gestational age at delivery calculated using ultrasound scan or last menstrual period [16].

Persistent infections were defined as follows: microscopic (beginning of infection)-microscopic (end of infection), microscopic-submicroscopic, submicroscopic-microscopic, and submicroscopic-submicroscopic infections.

**Figure 1.**
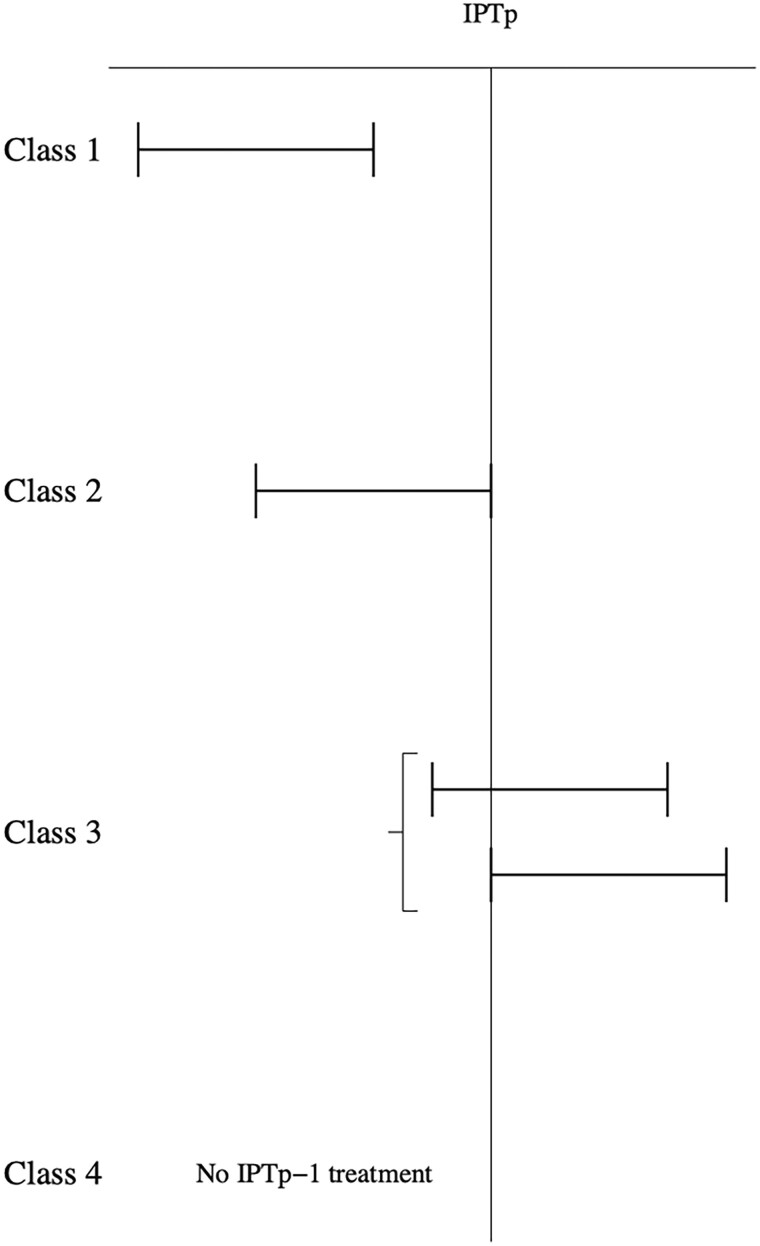
The relation between the occurrence and timing of a persistent infection (PI) and the first dose of IPTp (IPTp-1). Class 1: PI that begins and ends before the first dose of IPTp-1. Class 2: PI that begins before IPTp-1 and ends on the same day as IPTp-1. Class 3: PI that begins (before OR on the same day as IPTp-1 OR after the date of IPTp-1) AND ends after IPTp-1. Class 4: No IPTp-1 treatment.

The relation between the occurrence and timing of a persistent infection and the first dose of IPTp (IPTp-1) was investigated. For that purpose, persistent infections were classified in 4 classes based on the time of IPTp-1 (Figure 1), before, during or after each persistent infection.

Class 1: Persistent infections that begin and end before the first dose of IPTp-1.

Class 2: Persistent infections that begin before IPTp-1 and end on the same day as IPTp-1.

Class 3: Persistent infections that begin (before OR on the same day as IPTp-1 OR after IPTp-1) AND end after IPTp-1.

Class 4: No IPTp-1 treatment.

The maximum number of persistent infections during the pregnancy was 2. We have therefore assessed the timing of IPTp-1 related to the occurrence of the first persistent infection on the 1 hand and the occurrence of the second persistent infection on the other.

The analysis was performed using R studio.

## RESULTS

The main characteristics of women included in the present analysis are presented in Table 1. Our study population consisted of 130 of a total of 411 pregnant women included and followed up in the RECIPAL study.

**Table 1.** Characteristics of the Women of Reproductive Age and Pregnant Women Included in the RECIPAL Project, Benin, 2014–2017

Characteristics	Women Included in the Present Analysis (N = 130)	Final Cohort Pregnant Women (N = 411)
General characteristics		
Subdistrict, n (%)	…	…
Sô-Ava	110 (84.6)	132 (32.1)
Vekky	…	138 (33.8)
Houedo	…	33 (7.8)
Akassato	20 (15.4)	108 (26.3)
Age (y)	…	…
Mean (± SD)	26.41 (5.07)	26.8 (5.0)
Education, n (%)	…	…
Illiterate	99 (75.6)	290 (70.6)
Gravidity, n (%)	…	…
Primigravidae	13 (9.9)	33 (8.0)
Multigravida	118 (90.1)	378 (92.0)
Preconceptional characteristics	…	…
Body mass index, n (%)	…	…
<18.5 kg/m²	13 (9.9)	40 (9.7)
18.5‒24 kg/m²	85 (64.9)	265 (64.5)
≥25 kg/m²	33 (25.2)	106 (25.8)
Anemia, n (%)^a^	…	…
Yes	79 (60.1)	221 (53.8)
Microscopic infection, n (%)^b^	…	…
Yes	11 (8.5)	24 (5.9)
Gestational characteristics	…	…
Gestational age at first ANC visit (weeks)	…	…
Mean (± SD)	6.8 (2.31)	6.9 (2.5)
Number of ANC visits (scheduled)	…	…
Median (min-max)	7 (1–8)	6 (1–8)
Duration of the pregnancy (weeks)^c^	…	…
Mean (± SD)	39.5 (0.16)	39.0 (0.14)
HIV status (%)	…	…
Positive	2/130 (1.5%)	6/411(1.5%
Number of IPTp doses, n (%)	…	…
0 dose	20 (15.4)	…
1 dose	24 (18.5)	…
2 doses	71 (54.6)	…
3 doses	15 (11.5)	…
≥2 doses^d^	…	212 (54.0)

Abbreviations: ANC, antenatal care; IPTp, intermittent preventive treatment in pregnancy; PCR, polymerase chain reaction; RECIPAL, REtard de Croissance Intra-uterin et PALudisme; TBS, thick blood smear.

Compared to the 281 women not included in this study, the selected 130 women were significantly more likely to be infected with malaria during pregnancy (*P ≤* .001) and had higher coverage of IPTp (*P ≤* .001).

^a^According to World Health Organization thresholds (12 g/dL for nonpregnant women and 11 g/dL for pregnant).

^b^We have characterized each infection as submicroscopic infections, which were defined as TBS-negative/PCR-positive infections while microscopic infections were defined as TBS-positive/PCR-positive infections.

^c^Results based on the 287/411 and 112/130 women who delivered in the study maternity clinics.

^d^Results based on the 395/411 women included in the RECIPAL study for whom IPTp coverage was recorded.

By design, all studied women had at least 2 positive PCRs. We noticed that almost 40% of women in this study had 3 or more positive PCR infections during their pregnancy.

### Descriptive Analysis of Genotypical Data

**Figure 2.**
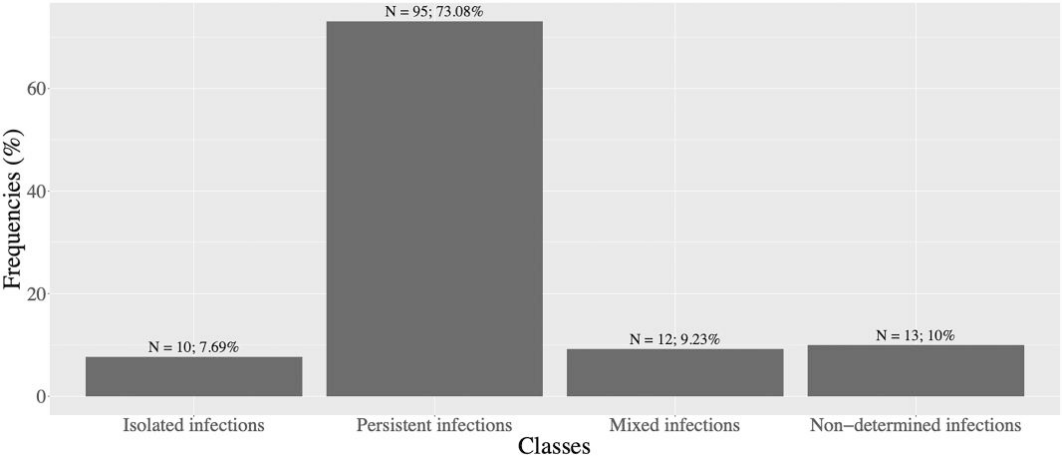
Classification of women depending on the type of infection identified by *msp-2* genotyping method: isolated infections, persistent infections, mixed of persistent and isolated infections, nondetermined infections (no data obtained).

We classified women based on the type of infection identified by *msp-2* genotyping method and the data are represented in Figure 2. We observed that 73.1% of women had only (exclusively) persistent infections (identical *msp-2* genotypes in all studied time points), 7.7% of women had only new infections (isolated infections) throughout pregnancy, and 9.2% of women had a mixture of persistent and isolated infections. For 13 of 130 women (10.0%), genotype data could not be obtained.

Among 130 pregnant women, 107 (82.3%) had at least 1 persistent infection during their pregnancy; among these 107 women, 101 (94.4%) had 1 persistent infection, whereas only 6 (5.6%) women were infected with 2 persistent infections. A total of 113 persistent infections were documented.

**Figure 3.**
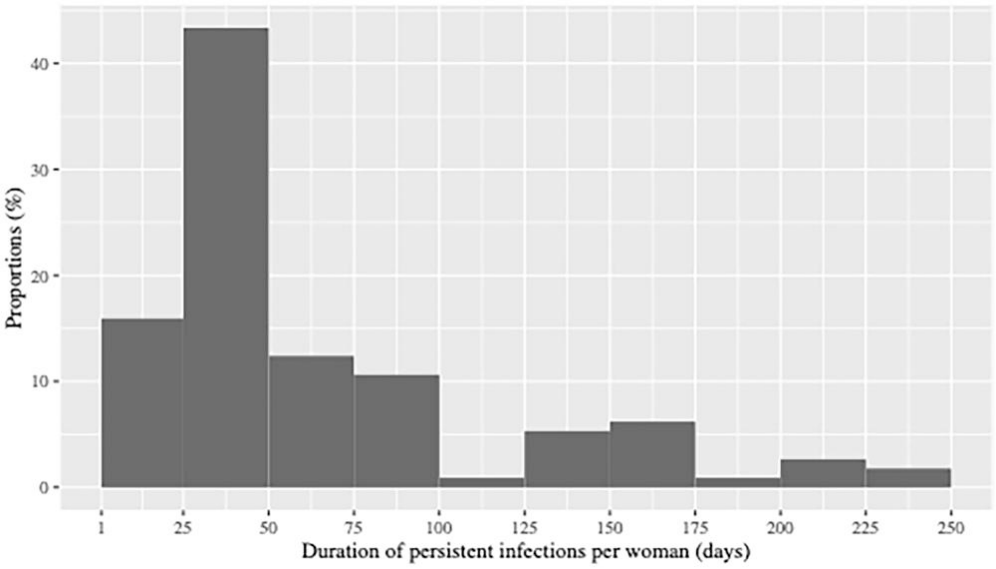
Distribution of the duration of persistent infections among women during pregnancy.

Figure 3 shows the distribution of the duration of persistent infections among studied women. It varied between 2 and 250 days with more than 50% of persistent infections under 42 days.

During pregnancy, the median time spent with 2 persistent infections was 25.4% of the total duration of the pregnancy and with 1 persistent infection, it was 18.4%.

Based on an arbitrary total duration of 39 weeks of pregnancy, these proportions correspond to 9.9 weeks and 7.2 weeks, respectively, for 2 persistent infections and 1 persistent infection.

**Figure 4.**
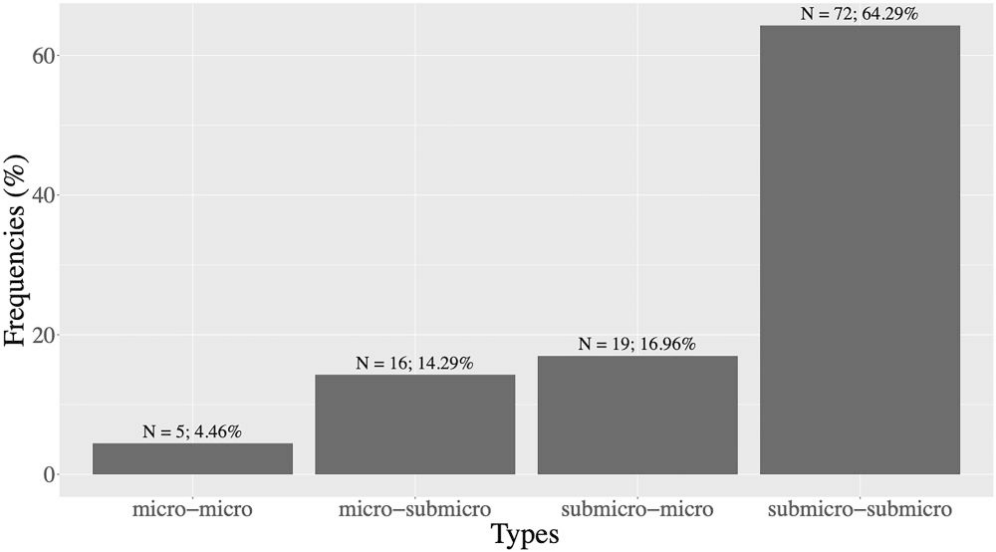
Characterization of persistent infections during pregnancy. Persistent infections are classified in 4 types: microscopic (beginning of infection)-microscopic (end of infection), microscopic-submicroscopic, submicroscopic-microscopic, and submicroscopic-submicroscopic infections.

Figure 4 shows the characterization of persistent infections during pregnancy. The frequency of each type is as follows: 64.3% of persistent infections contracted by women were submicroscopic-submicroscopic, 31.2% were a mixed infection (microscopic-submicroscopic), whereas 4.5% of persistent infections were bi-microscopic.

**Figure 5.**
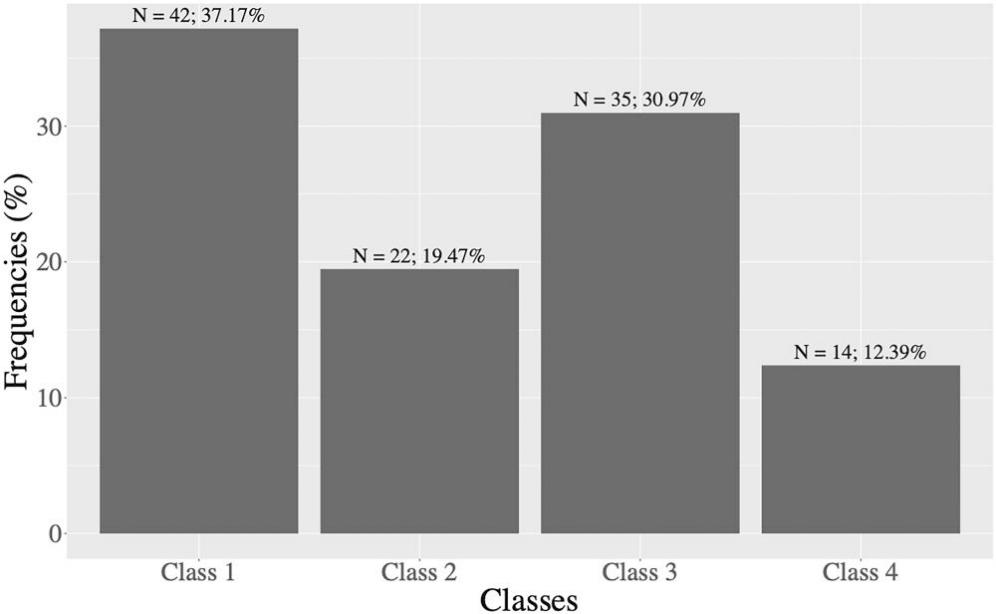
Persistent infections sub-classes according to IPTp first dose: Class 1: Persistent infections that begin and end before the first dose of IPTp-1. Class 2: Persistent infections that begin before IPTp-1 and end on the same day as IPTp-1. Class 3: Persistent infections that begin before, on the same day as IPTp-1 or after IPTp-1 AND end after IPTp-1. Class 4: No IPTp-1 treatment.

Figure 5 shows the persistent infections according to the IPTp first dose. We have noticed that 37.17% (n = 42) of the persistent infections were in the class 1 (starting and ending before IPTp-1); 19.47% (n = 22) of the persistent infections were in class 2 (ending at IPTp-1); the proportion of persistent infections belonging to class 3 (ie, starting before/at the time of or after IPTp-1 and persisting after IPTp1) was 30.97% (n = 35). Among them, approximately 20% (8/35) persisted despite the use of IPTp1, or even IPTp2 when women received it; the 27 other infections occurred 1 to 3 months after IPTp2 treatment, ended at the time of IPTp2 or occurred after the single first dose of IPTp. Finally, 12.39% (n = 14) of persistent infections throughout the pregnancy were contracted by women who had not received IPTp dose until delivery (class 4). These 14 infections occurred in a specific group of women with a very short duration of pregnancy. With the exception of 3 women who were followed up to 32, 36, and 40 weeks' gestation, the median duration of their follow-up was 16 weeks' gestation.

## DISCUSSION

The objective of the present study was to describe the dynamic of submicroscopic and microscopic malarial infections during pregnancy until delivery based on the genetic polymorphism of *P falciparum* infections in a context of high IPTp coverage. For that purpose, parasite genotyping was performed to distinguish persistent infections (identical genotype profiles of infection) from de novo infections (different genotype profiles of infection) during the pregnancy, before and after IPTp-SP.

Our team has shown in recent studies that a pregnant woman with a submicroscopic infection in the first or second trimester of pregnancy was significantly more susceptible to infection in the following trimester [23], suggesting the existence of persistent infections. Our results confirm this hypothesis showing that among infected women enrolled in the study, more than 70% had exclusively persistent infections throughout their pregnancy and until delivery, whereas only 8% of them had exclusively new infections. This means that most of pregnant women remain infected with the same *Falciparum* genotypes throughout their pregnancy. We have also shown that persistent infections can start before the pregnancy and remain until the first months of pregnancy [22].

We also show that a considerable part of these persistent infections is submicroscopic. This agrees with a recent study that assessed the dynamics and determinants of submicroscopic infections throughout pregnancy [23] and a meta-analysis study conducted by van Eijk et al. in 2023 [24]. Submicroscopic *P falciparum* infections during pregnancy are very common but rarely have been studied. Their impact must be assessed in each specific region because they depend on malaria transmission intensity and stability, maternal age, and parity. In endemic areas, submicroscopic infections are hardly detected and rarely treated by antimalarial drugs. They can therefore survive in the blood and play an important role in the transmission of the infection from human to the mosquito [25]. The high percentage of persistent submicroscopic infections in our study (64.3%) may be due to a high proportion of multigravida women (90.1%) and to their already built immunity against *P falciparum* infection that contributes to the clearance of the parasites throughout pregnancy. The consequence of this immunity is a very low parasitemia represented by the submicroscopic infections in these women. Because of a very few numbers of primigravidae in this study, we were not able to assess the persistence of submicroscopic infections in this specific group.

In our study, most of the infections were persistent and not de novo. This observation suggests that 1 dose IPTp-SP treatment during the pregnancy may not clear up the infection. Our study showed that nearly 20% of the persistent infections were not cleared either by IPTp1 or IPTp2. The failure of IPTp-SP treatment to clear the malaria infection can be explained by the widespread SP resistance in countries where SP had once been used as a first-line treatment. Previous studies have shown that mutations in *P falciparum* genes *dhfr* and *dhps* leads to parasite resistance to SP and decreases the efficacy of the treatment [26, 27]. It has also been shown that resistance to SP may be a major contributing factor to the levels of submicroscopic carriage in pregnant women because more than 60% of the submicroscopic isolates comprised at least a *dhps* mutation (A437G and/or A581G) [27]. In a recent systematic review in which the prevalence of K540E and A581G mutations in 294 surveys of infected humans across Africa from 2004 through the present was mapped, it has been observed that both K540E and A581G mutations increased in prevalence and frequency in 60% of areas after 2008 [28]. Moreover, a study in Benin showed that most parasite isolates (∼90%) carried the triple mutant *pfdhfr* in association with the single *pfdhps* mutation [29]. A similarly high prevalence of *pfdhfr/pfdhps* mutations (85%) before any administration of IPTp-SP was reported in Southern Benin in 2011 [30].

We used a fragment-analysis method to identify all the *P falciparum* genotypes within isolates and to quantify their proportions. We had used this methodology in a recent work in the RECIPAL project [26]. The strength of this study is the use of *msp-2* which is a highly polymorphic marker that provides a higher sensitivity in the detection of *P falciparum* genotypes compared to less polymorphic markers such as *Glurp*, *Msp-1* usually used in genotyping studies. We assume that the use of fluorescent primers and the fragment analysis method is an added value since it makes this genotyping method more sensitive and informative [22]. This approach consistently identifies minority genotypes with proportions as low as 0.5% in the patient isolate, compared to previously available molecular methods [31–33]. The true value of this method has already been evaluated in clinical settings in previous studies [15, 20, 22, 34]. The choice of this highly sensitive method is crucial for the detection of maximum number of genotypes specially in submicroscopic samples, which is the key topic of this study.

However, our study has some limitations that must be acknowledged. Our study population consisted in 130 selected women of 411 enrolled in the RECIPAL study, with at least 2 successive PCR-positive infections during pregnancy, which occurred within a delay of 2 months. Moreover, although most women in RECIPAL study had monthly antenatal care visits, the duration of the infections is depending on the schedules of these visits during the pregnancy in the selected women. These 2 parameters could have influenced our observations concerning the duration of persistent infections, duration of the time spent with an infection during pregnancy, and the effect of IPTp on these infections.

In this study, we evidenced that persistent low-density/submicroscopic infections are common during pregnancy with a noticeable proportion of them persisting despite the use of IPTp-SP. These infections, which are likely asymptomatic, remain untreated and can persist throughout the pregnancy in the absence of a strategy to prevent or treat them effectively. IPTp is a pragmatic strategy that aims to clear and prevent all potential malarial infections regardless of their parasite density. Moreover, SP treatment retains certain benefits (particularly regarding unfavorable pregnancy outcomes) even in areas of resistance.

In high-transmission areas, this strategy has proven to be of greater interest compared to the Intermittent Screening and Treatment strategy consisting in treating women identified as infected only [35]. Making the administration of a higher number of IPTp-SP doses possible and effective using community delivery approaches [36, 37] or using new antimalarial combinations for IPTp such as dihydroartemisinin-piperaquine [38] could contribute to limit the persistence of these infections and their deleterious effects on the mother and the fetus [39]. Finally, persistent infections have been shown to start as early as in the first trimester, or even in the preconception period [22]. Preconceptional interventions such as malaria vaccine for childbearing age women might also contribute to better prevent these infections [40].

## Supplementary Material

ofae762_Supplementary_Data

## Supplementary Data

Supplementary materials are available at *Open Forum Infectious Diseases* online. Consisting of data provided by the authors to benefit the reader, the posted materials are not copyedited and are the sole responsibility of the authors, so questions or comments should be addressed to the corresponding author.

## Notes

***Acknowledgments.*** We are extremely grateful to all women who took part in this study, the midwifes, nurses, and community health workers for recruiting and following them, and the whole RECIPAL team, including engineers and technicians, who were a key in obtaining high-quality data and samples.

***Author contributions.*** V.B., G.C., and S.J.-G. contributed to conceptualization, data interpretation, and writing the original draft. N.F. contributed to data collection. M.A. contributed to data collection and study management. N.T.N., A.M., L.C., V.S.-H., and S.J.-G. contributed to investigation. A.M. contributed to supervision. R.D. contributed to statistical analysis. V.B., G.C., S.J.-G., X.C.D., A.M., N.F., N.T.N., and A.M. contributed to review and editing the final draft.

***Financial support*.** This work was supported by the Bill and Melinda Gates Foundation [OPP1169555]. The RECIPAL project was supported by the French Agence Nationale de la Recherche [ANR-13-JSV1-0004, grant 2013] and the Fondation Simone Beer under the auspices of the Fondation de France [00074147, grant 2017]. Valérie Briand was in receipt of grants.

***Patient Consent Statement.*** The design of the work has been approved by the Ethics Committee of the Institut des Sciences Biomédicales Appliquées and the Ministry of Health in Benin. Before recruitment, the patient's written consent was obtained.
