# Correction to: Dynamics of Persistent Submicroscopic and Microscopic *Plasmodium falciparum* in Pregnant Women Under Intermittent Preventive Treatment: A Study Cohort in Benin

**DOI:** 10.1093/ofid/ofaf071

**Published:** 2025-02-13

This is a correction to: Sayeh Jafari-Guemouri, Robinson Dégbègni, Laura Courtois, Manfred Accrombessi, Achille Massougbodji, Xavier C Ding, Nicaise Tuikue Ndam, Atika Mama, Nadine Fievet, Véronique Sarrasin-Hubert, Gilles Cottrell, Valérie Briand, Dynamics of Persistent Submicroscopic and Microscopic *Plasmodium falciparum* in Pregnant Women Under Intermittent Preventive Treatment: A Study Cohort in Benin, *Open Forum Infectious Diseases*, Volume 12, Issue 1, January 2025, ofae762, https://doi.org/10.1093/ofid/ofae762

In the originally published article, the eleventh author was incorrectly listed as Gilles Cotrell. This is now corrected to read: “Gilles Cottrell”.

