# Supplementary material for: Dynamics of Persistent Submicroscopic and Microscopic Plasmodium falciparum in Pregnant Women Under Intermittent Preventive Treatment: A Study Cohort in Benin
Source: Open Forum Infect Dis. 2025 Jan 6;12(1):ofae762. doi: 10.1093/ofid/ofae762 (PMC11739809; doi:10.1093/ofid/ofae762)
Supplement: ofae762_Supplementary_Data [file ofae762_supplementary_data.pdf]

Supplementary table summarizing the number of *msp-2* gneotypes detected at various time points in 130 pregnant women from RECIPAL project, included in this study

Plasmodium falciparum DNA was genotpyed from positive infections that were detected during scheduled visits (AnteNatalConsultations ANC 1 to ANC 8), unsecheduled visits (U1, U2) and Delivery time points. A fragment-analysis method was used to determine and enumerate *P. falciparum* genotypes for 130 women at different time points. ND (Non Determined) : *msp-2* genotyping has failed for these time points, or not enough DNA for genotyping. Empty boxes are time points not selected for *msp-2* genotyping.

Women ID	ANC 1	ANC 2	ANC 3	ANC 4	ANC 5	ANC 6	ANC 7	ANC 8	Delivery	U1	U2
AH063	5	2							2	1	
AH109	4	5									
NA034	2	2									
LA021	4	4									
NA025	9									9	
NA050	18	18									
RE051	15								7		
SZ088	3	10									
SZ116	2	3									
NA003			14	14							
NA024							3		2		
RE058						16			15		
AH048	2	3									
RE015		2	11								
AH081				5	13						
AH060							9		9		
RE010	9	7									
RE024	7	7									
SZ019	8	10									
SZ086		1									
SZ093		8	4								
EG004				6	7						
EG101	6	8									
SZ012		9	13								
SZ048				9	10						
SZ078		7	7								
SZ073	8	7									
RE117						8	7				
NS086	10	10									
SZ143	10	9									
SK107			8	7							
SK029						5			14		
AH233		7	7								
SK060							15		16		
LA020		10	10								

Women ID	ANC 1	ANC 2	ANC 3	ANC 4	ANC 5	ANC 6	ANC 7	ANC 8	Delivery	U1	U2
SK032			7	7							
AH170	9	15									
NA127		12	13								
EG100				18	18						
SZ062			6	6							
NA001			11	12							
AH148					20	19					
EG105						6			6		
RE021									2	7	
NA085	5									5	
RE025			3	4							
AH037									2	3	
SZ007	19	7									
RE103		10								19	
SK023					12	ND					
AH314			16	19							
AH161						15	15				
AH261									ND	ND	
SK084		ND	ND								
NS048	ND										
NA014	ND	ND									
RE045				ND	6						
SZ061	ND	ND									
SZ052		ND	ND								
AH003	4	6								6	
AH038	2	2	5								
AH144			6							6	6
RE108	19	15	17								
SZ111	21	12	20						7		
SZ030	7	9	9								
SZ101		3	3		4				12		
AH026	2	2	2								
NA007	16	13	16	16	17	17	17		17		
AH139	11	11	12								
AH054	10	7	9								
RE105	12	6								12	
SZ094	11	12	18								
EG014		3	5	3							
EG085	8	8	9	7							
EG038	5	5			5	5	6		6		
EG027			4	5	5						
SZ135	14	14	14								
SK072	9	10	9								
SK096	7	1	7								
NA122	7	7	7								

Women ID	ANC 1	ANC 2	ANC 3	ANC 4	ANC 5	ANC 6	ANC 7	ANC 8	Delivery	U1	U2
AH174	6	6	6								
NA044						9	12		11		
NA162					7	6				7	
SK043	7	7	5								
SZ077						2	10		11		
SZ036	6	5		2							
RE107		10	10	11							
LA043	5		8	8							
AH029		4	7	7	7						
LA024	11	13	ND								
NA066	10	10								ND	
LA028	ND	ND	ND								
NS003		7	ND	ND							
AH096	ND	ND	15								
SZ050		ND	10	9							
NA126	14	14	ND								
LA107	ND	11	11								
RE034	ND	5	10								
AH164	ND	ND									
AH146	5	2	4							4	
NA088				17	14	15	11				
RE078			20	21	19	17					
SZ112	5	5				4			3		
AH115	18	16								18	20
RE092	11	7	5							6	
RE020						8	11	9	7		
AH089		7	8							14	13
RE096	7	6	9	8							
EG025	12	12	15	16							
EG095	9	10	10	8							
NA102	10	13	13	13		13		4		4	
NA121	6	3	10	10							
SZ084		9		10						10	10
AH240						13	13		ND		10
LA026	6	6	14	15							
AH169		15	ND			18			17		
NA143	ND	ND	ND	ND							
AH142	12	12	11	10	8					ND	
NA041			2	5	4	2			5		
NA009	14	16	14	12	20						
EG017	13	12				10	4		9		
NA098	8	9	8	8	8						
SZ137	15	16	16	15	15						
NA011	15	ND	ND		19	5					
AH104	7	6	8	4	7					8	
EG012	10	9		12	12	12				12	

Women ID	ANC 1	ANC 2	ANC 3	ANC 4	ANC 5	ANC 6	ANC 7	ANC 8	Delivery	U1	U2
LA045	19	18	ND	14	ND	17					
AH097	12	8	16	16	9	6				11	
NA013	7	3		5	11		11		ND	4	
